# Immunomodulatory Effects of Flavonoids: Possible Induction of T CD4+ Regulatory Cells Through Suppression of mTOR Pathway Signaling Activity

**DOI:** 10.3389/fimmu.2019.00051

**Published:** 2019-01-31

**Authors:** Aysooda Hosseinzade, Omid Sadeghi, Akram Naghdipour Biregani, Sepideh Soukhtehzari, Gabriel S. Brandt, Ahmad Esmaillzadeh

**Affiliations:** ^1^Department of Immunology, Faculty of Medicine, Shahid Sadoughi University of Medical Sciences, Yazd, Iran; ^2^Students' Scientific Research Center, Tehran University of Medical Sciences, Tehran, Iran; ^3^Department of Community Nutrition, School of Nutritional Sciences and Dietetics, Tehran University of Medical Sciences, Tehran, Iran; ^4^Department of Nutrition, School of Health, Shahid Sadoughi University of Medical Sciences, Yazd, Iran; ^5^Department of Pharmaceutical Science, University of British Columbia, Vancouver, BC, Canada; ^6^Department of Chemistry, Franklin & Marshall College,, Lancaster, PA, United States; ^7^Obesity and Eating Habits Research Center, Endocrinology and Metabolism Molecular-Cellular Sciences Institute, Tehran University of Medical Sciences, Tehran, Iran; ^8^Department of Community Nutrition, Food Security Research Center, School of Nutrition and Food Science, Isfahan University of Medical Sciences, Isfahan, Iran

**Keywords:** flavonoids, mTOR, autoimmunity, metabolism, T regulatory cells

## Abstract

The increasing rate of autoimmune disorders and cancer in recent years has been a controversial issue in all aspects of prevention, diagnosis, prognosis and treatment. Among dietary factors, flavonoids have specific immunomodulatory effects that might be of importance to several cancers. Over different types of immune cells, T lymphocytes play a critical role in protecting the immune system as well as in the pathogenesis of specific autoimmune diseases. One of the important mediators of metabolism and immune system is mTOR, especially in T lymphocytes. In the current review, we assessed the effects of flavonoids on the immune system and then their impact on the mTOR pathway. Flavonoids can suppress mTOR activity and are consequently able to induce the T regulatory subset.

## Introduction

Nutrition and metabolism play an important and undeniable role in public health. Although genes have specific importance in susceptibility to diseases, some environmental factors can affect a gene's ability to “switch on or off” (1). In fact, phenotypes are determined by a combination of genotypic and environmental factors (2). Diet is one of the environmental factors that could be considered in the prevention and treatment of several disorders (3), including some autoimmune diseases as MS (3, 4) and type I diabetes (4). Chemo-preventive effects of diet on cancer (5, 6) and autoimmune diseases have also been reported (7, 8).

The immune system plays a critical role in protecting the human body from infectious diseases and cancer. Its two main contributors include innate and acquired immunity responses. The most important feature of innate immunity is its lack of specific recognition. This arm of the immune system responds to all pathogens regardless to their nature (9). In contrast to innate immunity, acquired immunity recognizes pathogens specifically and responds to each pathogen according to its nature. Innate immunity is composed of immune and non-immune components, whilst acquired immunity has only immune elements. The major functions of the acquired immune system rely on immune cells, mainly B and T lymphocytes that recognize pathogens based on their antigenic receptors and respond in different ways. B cells produce antibodies to block pathogen activity and opsonize them for phagocytes. T cells are divided into two subsets: T cytotoxic cells (T CD8^+^) which kill cancer cells directly and T helper cells (T CD4^+^) that secrete cytokines and mediators that orchestrate other cells such as B lymphocytes and macrophages (9–11). Th cells secrete a wide range of cytokines, which can direct the type of antibodies produced by B cells and also are able to activate and polarize monocytes and macrophages. In light of this, Th cells play a central role in the immune system (12).

**Figure 1:**
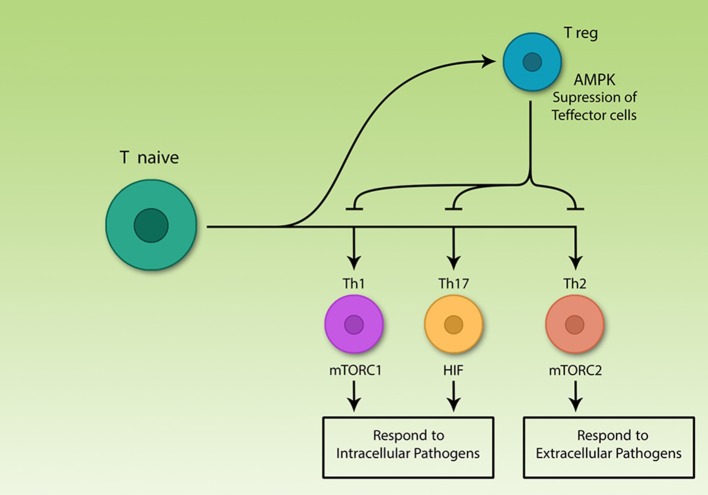
The central role of T lymphocytes in acquired immunity. Acquired immune cells, notably lymphocytes, recognize pathogens specifically and respond to them, according to their nature. T helper lymphocytes secrete various cytokines which are able to activate different immune cells including acquired and innate cells. The various Th subsets rely on different signaling pathways, but all effector subsets upregulate glycolysis. Among differentiated Th cells, T regulatory cells are the only non-glycolytic cells; effector subsets use different signaling molecules, however, all are glycolytic.

One of the research areas of immunometabolism is to study the effect of different metabolites on Th cell differentiation. Many researches have focused on the effects of glucose (13), oxygen (14), salt (3), fatty acids (1, 15–17), vitamins (18–20), and amino acids (21, 22) on mechanisms involved in Th cell differentiation. The importance of Th cell differentiation becomes clear when we consider uncontrolled T_eff_ activation against self-antigen that triggers an immune response, through which damages self-tissue and interrupts some organ functions. One of the important dietary factors studied in terms of immunomodulatory effects are polyphenols and their effects on the level and composition of immunoglobulins, inflammation, immune cell population content and also their antioxidant effects on cancer cells have been investigated (23–25). Several studies have reported immunomodulating effects of polyphenols (26, 27). However, it remains unknown whether Th cells and changes in the ratio of inflammatory/regulatory cells can mediate such effects of polyphenols. Inflammatory and regulatory subsets of Th cells have different metabolic demands. In inflammatory subsets of Th cells, the mTOR pathway is activated. This pathway promotes glycolysis to supply their energy needs. This pathway is inactive in regulatory cells. If the mTOR pathway is suppressed in inflammatory subsets, they differentiate into the regulatory subset. Therefore, activation or suppression of the mTOR pathway determines Th cell differentiation into inflammatory and regulatory subsets (Figure 1). It is still unknown if flavonoids can suppress mTOR function and consequently induce T_reg_ subsets. The current study summarizes the effects of flavonoids on the immune system and subsequently the role of specific pathways like PI3k/Akt/mTOR on immunomodulation and possible effects of polyphenols on this pathway.

Th cell subsets are classified into Th1, Th2, Th17, and T_reg_. Th1, Th2, and Th17 are effector subsets and trigger immune response to different pathogens (13, 28, 29) while T_reg_ cells restore homeostasis by suppression of T_eff_ cell function after termination of immune response (30, 31). Precise function of the immune system is important for correct immune response, otherwise two pathological types of responses might occur. On the one hand, if the immune system fails to detect pathogens or cancer cells for any reason, the risk of developing disease increases (32). On the other hand, if the immune system cells by mistake identify self-antigens as foreign agents, the response of killer cells and/or antibody-producing cells interfering with cytokine levels, can result in serious damages to the body (33).

Currently, there is only sketchy understanding of the factors and mechanisms involved in autoimmunity. The loss of self-tolerance is one of the important causes of disease. Although the mechanism of loss of immune tolerance is not yet fully understood, some behaviors like smoking may interfere with tolerance and lead to autoimmunity disorders (34). For this reason, other environmental factors like diet also play a critical role in immune tolerance failure. In normal immune function, both central and peripheral tolerance mechanisms do not allow the immune system to respond against self-antigens (14). T_reg_ cells are one of these tolerance mechanisms, since T_reg_ cell suppress T_eff_ cells and consequently is able to block unwanted prolonged immune response (35). Depending on the types of immune cells involved, symptoms, and treatment of this disease differ.

In addition to the possible role of diet in immune tolerance failure, this question arises whether the immune system and various metabolites can interact with each other. In fact, not only metabolites can affect the immune system, but also the function of the immune system affects metabolic tissues. This mutual interaction is referred as immunometabolism. Interestingly, malnutrition has a negative impact on immune function. But what is more, oversupply of metabolites is destructive as well (36).

As mentioned above, flavonoids generally have immunomodulatory effects on the immune system. To understand it better, at first we look at the effects of four common flavonoids on immune system. Here we get general information about the effects of flavonoids on different compartments of immune system; function of innate and acquired immune cells, antibody production, cytokine secretion, and nuclear transcription factor activity. The entire data suggests that flavonoids can suppress pro-inflammatory immune response.

## Flavonoids and Their Effects on Immune System

Flavonoids are considered as plant secondary metabolites that numerous pharmacological functions are attributed to them including antioxidant, anti-mutagenic, antibacterial, anti-angiogenic, anti-inflammatory, anti-allergic, enzyme modulation, and anti-cancer (37, 38). They are defined as phytochemicals which exist either as free aglycones or glycosidic conjugates (39). Flavonoids are polyphenolic with a wide range of structures (37). Based on this diversity, they are categorized mainly into flavones, flavanols, isoflavones, flavonols, flavanones, flavanonols, and chalcones (39). The diverse structures of flavonoids have resulted in many properties including anti-cancer and anti-inflammatory effects (37, 39, 40). Recently, it has been shown that flavonoids can affect immune system response and might have immune-modulator effects.

### Quercetin

Quercetin is an abundant polyphenol in nature. It is an aglycone form of a number of other flavonoid glycosides such as rutin and quercitrin which can be found in variety of foods and plants, including apples, berries, Brassica vegetables, capers, grapes, onions, shallots, tea, and tomatoes, as well as different seeds, nuts, flowers, barks, and leaves (41, 42). Biosynthesis of Quercetin starts with phenylalanine in plants (41). It has been shown that Quercetin can affect lipid and glucose metabolisms by reducing oxidative stress and enhancing β-oxidation (43). In addition, some studies have examined the effects of Quercetin on the immune system. In an experimental study, dendritic cells (DCs) obtained from mouse bone marrow were treated by Quercetin. This flavonoid could effectively decrease the production of pro-inflammatory cytokines/chemokines and the expression levels of MHC class II and co-stimulatory molecules. These conditions inhibit the LPS-induced activation of DCs. Furthermore, endocytosis of DCs and the LPS-induced DC migration are decreased by Quercetin treatment (43). Quercetin also diminishes Ag-specific T cell activation by reducing the activity of LPS-stimulated DC's (44). In another experimental study, the effects of Quercetin-loaded micro-emulsion (QU-ME) were examined in a model of airway allergic inflammation. Mice received daily oral doses of QU-ME (3 or 10 mg/kg) over the course of 22 days. Compared with control group, QU-ME reduced inflammatory factors including IL-5 and IL-4. However, no change was observed in CCL11, IFN-gamma, and LTB levels. In addition, the nuclear transcription factor kappa B (NF-kappa B) activation, P-selectin expression and the mucus production in the lung were inhibited by oral treatment of QU-ME (41). In a study on peripheral blood mononuclear cells (PBMC) isolated from multiple sclerosis (MS) patients and from normal healthy subjects, Sternberg et al. showed that Quercetin decreased the proliferation of PBMC and modulated the level of IL-1beta and TNF-alpha released by PBMC in a dose-dependent manner. In this study, the modulation of TNF-alpha increased when Quercetin combined with human interferon-beta (IFN-beta) (45). In another mouse asthma model, Gupta et al. examined the potential of Quercetin to relieve asthma aggravation. This study revealed anti-asthmatic potential of Quercetin. Treatment with Quercetin significantly resulted in a reduction of specific immunoglobulin E (sIgE) production and anaphylaxis signs. Furthermore, Quercetin modulated the expression of Th2 cytokines including IL-4 and IL-5. These cytokines play a role in switching IgE class and suppressing the degranulation/secretion of different chemical mediators (PGD2, mMCPT-1 Cys-L, and TSLP) from activated mast cells (46). Other studies on the effects of Quercetin on the immune system showed inhibitory effects of Quercetin on cytotoxic lymphocyte function (47), IL-6 production in LPS-stimulated neutrophils (48), and anaphylactic contraction in guinea pig ileum smooth muscle (49). Moreover, it has been observed that Quercetin can regulate leukocyte biology with a stimulus-specific action and affects the balance of Th1/Th2 in a murine model of asthma (50, 51). Based on these findings, Quercetin has a potential role in modulating immune system responses.

### Luteolin

Luteolin (3′,4′,5,7-tetrahydroxyflavone) and its glycosylated form luteolin-7-glucoside (L7G) belong to the flavone subclass of flavonoids and are among the most common flavonoids present in aromatic plants and other plant-based foods mostly consumed in the Mediterranean diet. Also, It is well distributed in many medicinal plants and some common fruits and vegetables including green leafy plants such as parsley, sweet peppers and celery (52–54). Although, glycosylated forms are the most common in nature it has been reported that Luteolin is absorbed in the aglycone form only. Apart from the antioxidant and anticarcinogenic properties, other features as anti-inflammatory and anti-allergic have also been reported for Luteolin (55–58). In an experimental study, treatment of asthmatic models of rats by Luteolin over 8 weeks resulted in a reduction in the total cell count, neutrophil count, eosinophil count and levels of IL-4 in comparison to a control group (59). In another mouse study, the effect of Luteolin on experimental autoimmune thyroiditis (EAT) showed that Luteolin treatment decreased lymphocytic infiltration and follicle destruction in thyroid glands. In addition, Luteolin inhibited the interferon-γ-induced increase in cyclooxygenase 2, and the secretion of the pro-inflammatory cytokine tumor necrosis factor-α (60). In an experimental study on human and murine auto-reactive T cells, Verbeek et al. reported that luteolin was a strong inhibitor for both murine and human T-cell responses. In this study, T-cell proliferation, and antigen-specific IFN-gamma production were significantly reduced in response to luteolin treatment. In addition, luteolin appears to be a strong inhibitor of mast cell histamine secretion (61). Moreover, anti-bacterial and anti-parasite properties of luteolin have been reported in recent studies (62, 63). The effects of Luteolin on the immune system and inflammation have also been assessed *in vivo* (64). Topical application of *Reseda luteola* extract, which is high in Luteolin, was as effective as hydrocortisone in decreasing inflammation following skin irradiation with Ultraviolet-B light (64). Overall, it seems that luteolin has beneficial effects on the modulation of immune responses. However, the mechanisms of this action might be variable and are not clearly known. Further studies are needed to shed light on these mechanisms.

### Apigenin

Apigenin, or 40,5,7-trihydroxyflavone, is a common dietary flavonoid which is found in many fruits, vegetables, and herbs, such as orange, grapefruits, onion, wheat sprouts, parsley, celery, and chamomile tea (65, 66). Properties of Apigenin include anti-proliferative, anti-cancer antioxidant and anti-inflammatory activities (67). Apigenin exhibits anti-tumor effects by decelerating growth and inducing apoptosis through activation of pentose phosphate pathway-mediated NADPH generation in HepG2 human hepatoma cells, induction of apoptosis via the PI3K/AKT and ERK1/2 MAPK pathways, decreasing the viability, adhesion, and migration of cancer cells and modulating angiogenesis and metastasis (68). The effects of Apigenin on the immune system or modulation of immune responses have been assessed in recent studies. In an experimental study, Cardenas et al. reported Apigenin significantly modulated NF-κB activity in the lungs. This finding showed the ability of Apigenin to exert immune-regulatory activity in an organ-specific manner (69). In another study on models of rat colitis, administration of apigenin K, a soluble form of Apigenin, resulted in reduced inflammation as well as lower colonic damage scores and colonic weight/length ratio (68). In addition, administration of Apigenin K could normalize the expression of some colonic inflammatory markers [e.g., TNF-α, transforming growth factor-β, IL-6, intercellular adhesion molecule 1 or chemokine (C-C motif) ligand 2] (70). In another experimental study on asthma in mice, Li et al. reported that Apigenin administration (5 mg/kg or 10 mg/kg) inhibited OVA-induced increases in eosinophil count and also in Th17 cells. Therefore, Apigenin administration might effectively ameliorate the progression of asthma (71). Furthermore, it has been shown that Apigenin in combination with Quercetin and Luteolin has a protective effect on pancreatic beta-cells injured by cytokines during inflammation (72). The inhibitory effect of Apigenin on mast cell secretion has also been observed in recent studies (51). Apigenin combined with Luteolin are strong inhibitors for murine and human T-cell responses, in particular auto-reactive T cells (61). In sum, it seems that apigenin can be considered as a modulator of immune system.

### Fisetin

Fisetin (3, 3′, 4′, 7-tetrahydroxy flavone) is a type of flavonoid commonly found in plants like the smoke tree and numerous types of fruits and vegetables including strawberries, grapes, onions, and cucumbers (51, 73–75). Some properties of Fisetin include anti-cancer, anti-angiogenic, neuroprotective, neurotrophic, antioxidant, anti-inflammatory, anti-proliferative, and apoptotic effects (76). However, the powerful antioxidant property of Fisetin is due to the presence of phenolic hydroxyl group in the flavonoid structure (77). A few studies have examined the effects of Fisetin on the immune system. Song et al. assessed the immunosuppressive effects of Fisetin against T-cell activation *in vitro* and *in vivo*. Findings of this study showed that Fisetin significantly inhibited Th1 and Th2 cytokine production, cell cycle and the ratio of T CD4^+^/CD8^+^ cells *in vitro*. Furthermore, Fisetin suppressed mouse T lymphocytes through the suppression of nuclear factor kappa B activation and nuclear factor of activated T cells signaling in a dose-dependent manner. The *in vivo* finding showed that Fisetin also inhibited delayed-type hypersensitivity reactions in mice (76). One study on the effects of Fisetin on human mast cells (HMC-1) showed that Fisetin could down-regulate mast cell activation (73). In addition, two studies have reported that the anti-asthma properties of Fisetin are due to reduction of Th2 response as well as suppression of NF-κB (75, 78). In an experimental study using a mouse model of atopic dermatitis (AD), Kim et al. investigated the effects of Fisetin on AD-like clinical symptoms. They showed that Fisetin administration inhibited the infiltration of inflammatory cells including eosinophils, mast cells, and T CD4^+^ and T CD8^+^ cells. Furthermore, Fisetin was able to suppress the expression of cytokines and chemokines associated with dermal infiltrates in AD-like skin lesions. In a dose-dependent manner, Fisetin decreased the T CD4^+^ cell-induced production of interferon-gamma and interleukin-4, and in contrast, increased the anti-inflammatory cytokine such as interleukin-10 (79). Based on these findings, Fisetin is able to significantly affect immune system responses.

As mentioned, T CD4 ^+^ cells play a central role in orchestrating immune response. Moreover, while regulatory effects of flavonoids on T CD4^+^ have been observed, the exact mechanisms are under investigation. Here we elaborate why metabolism can play an important role in Th cells fate. What happens to metabolic machinery of Th cells when they get activated? Studies show that metabolic status of naive and activated Th cells is different, because of their different energetic demands.

## Metabolism of Th cells

Resting and naive Th cells don't need great amount of energy. Hence, their metabolic status is generally at baseline. These cells use autophagy and catabolism of fatty acids to supply their housekeeping demands (80). When these cells are activated, they undergo rapid and excessive clonal expansion. Activated Th cells use anabolism to synthesize different types of essential macromolecules for proliferation, which is highly energetically costly. In fact, activated Th cells switch from catabolism to anabolism, a process known as metabolic reprogramming (80).

**Figure 2:**
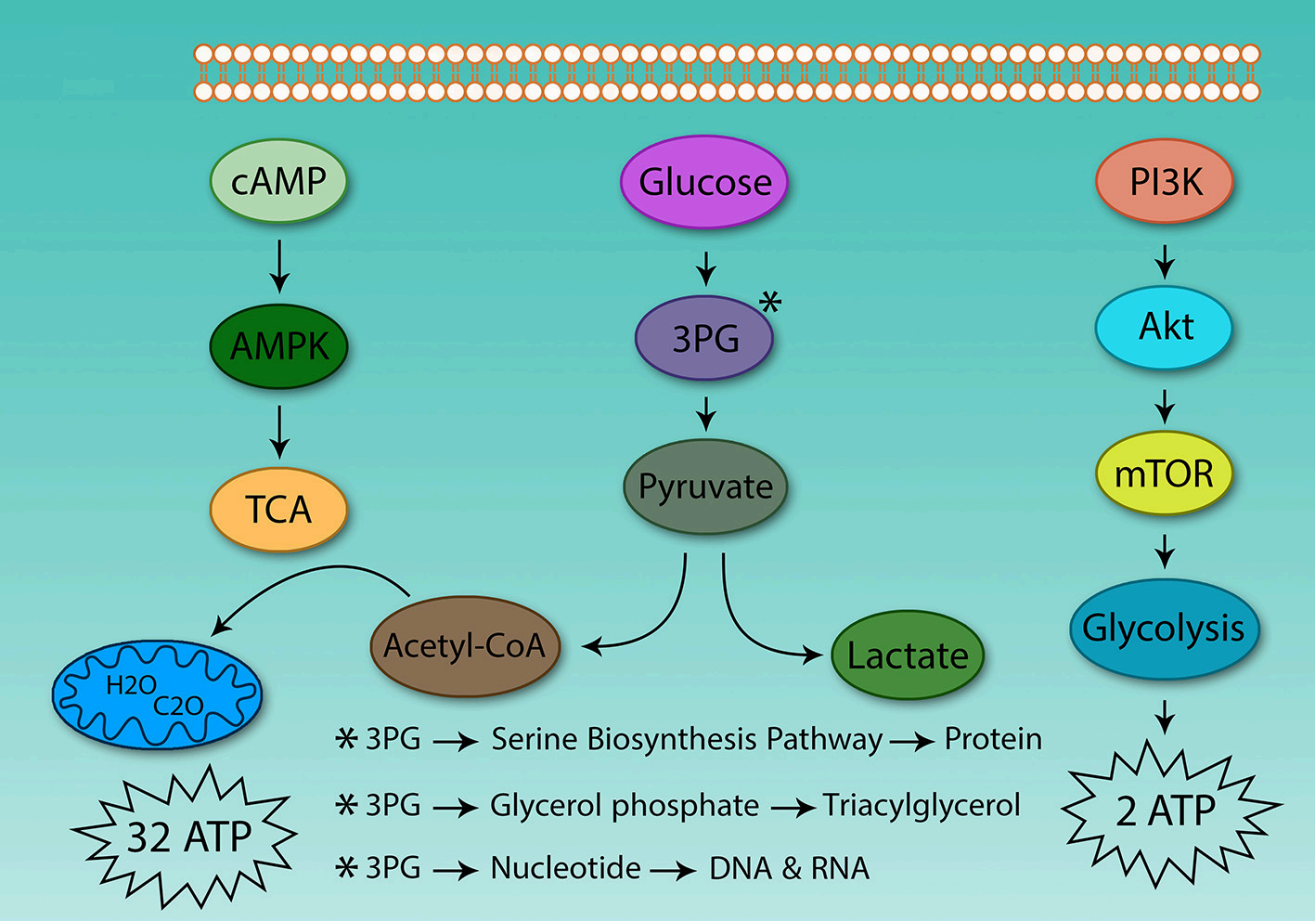
Differences between glycolysis and the tricarboxylic acid cycle. The energy obtained from glucose oxidation and glycolysis is very different, even in oxygen-activated Th cells which use glycolysis. The same situation also is observed in cancer cells. Because of this, the tumor environment is acidic. Activated Th and cancer cells use glycolysis to supply protein, DNA, and lipids to support proliferation.

The hallmark of Th cell metabolic reprogramming is the use of glycolysis in the presence of sufficient oxygen (81). If following activation, Th cells are not able to induce metabolic reprogramming, they become anergic, and are not able to respond to pathogens (82). Therefore, the metabolism of these cells plays a critical role in Th cell activation. However, the main question is why activated Th cells use glycolysis instead of TCA for ATP production? Why do they prefer to use a low yield pathway (2 ATP) instead of high-yield cycle (32 ATP)? Although clonal expansion requires energy, it also relies on protein, DNA, and lipid synthesis for cell size augmentation, for which glycolysis provides the energetic drive. Otto Warburg in 1931 found that cancer cells grow in acidic conditions, as they use glycolysis and produce lactic acid (Figure 2).

Below, we discuss how metabolism and immune signals are linked together. Some immunological signals are integrated into metabolic pathways. One of the most important pathways which plays key role in Th cell differentiation is PI3K/Akt/mTOR pathway. The activation status of this pathway is affected by different immunologic signals. PI3K/Akt/mTOR pathway promotes glycolysis and it is necessary to be increased as it activates glycolysis pathway significantly and also increases the expression of a range of proteins including enzymes and transporters. The PI3K/Akt/mTOR pathway mediates up-regulation of glycolysis and prepares cells for proliferation.

## mTOR; Mechanistic Target of Rapamycin

mTOR is a highly conserved molecule in mammalian cells (83), coded as a unique single gene but translated into two different proteins, mTOR complex 1 and 2 (mTORC1 and mTORC2) (82). These two complexes have different functions. Activation of mTORC1 results in enhancement of translation, cell size and lipogenesis in white fat tissue, whilst mTORC2 activation promotes glucose uptake in tissue, enhancement of glucose synthesis and reduction of gluconeogenesis in liver (83).

**Figure 3:**
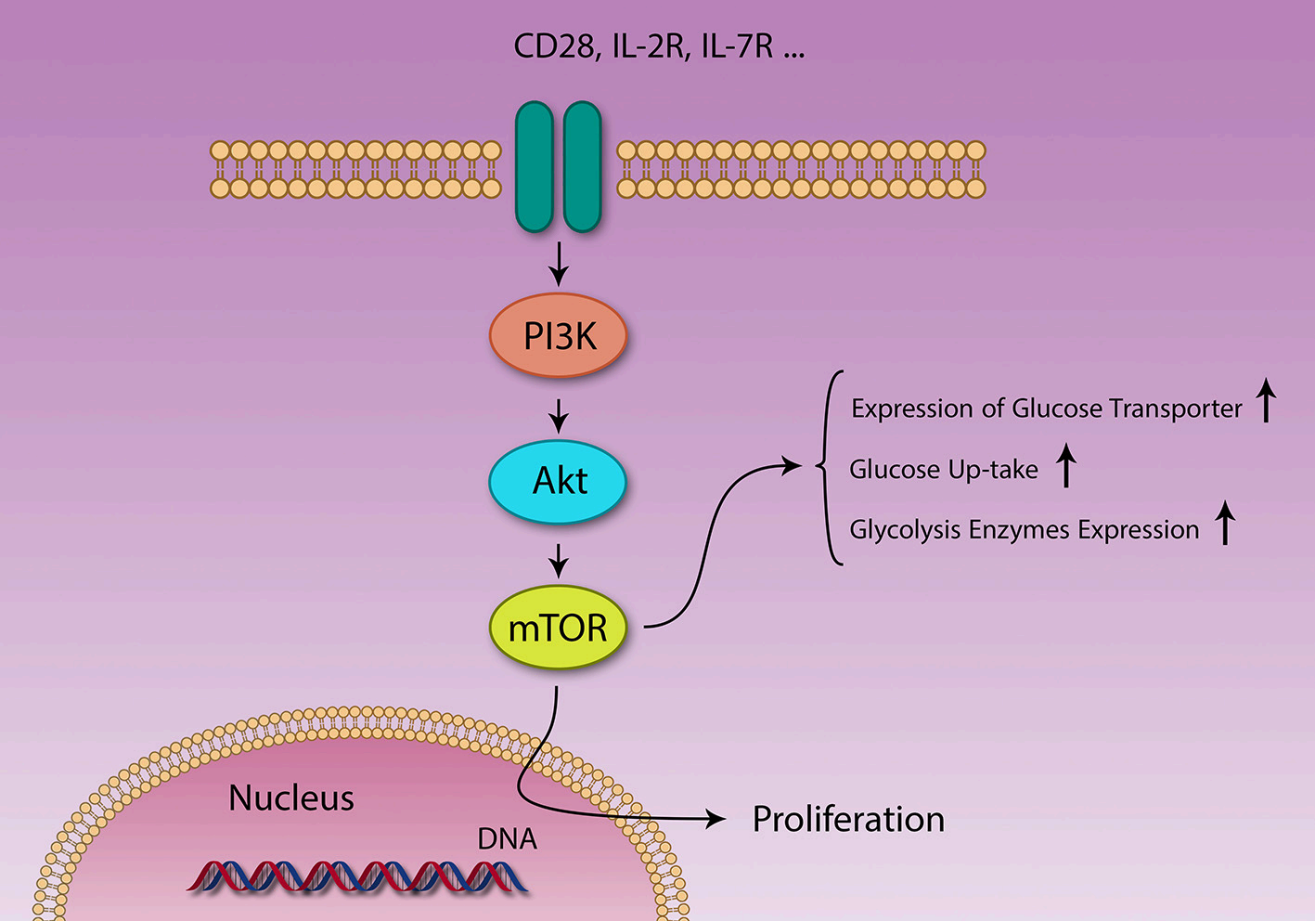
Different signaling pathways are activated by different stimuli. PI3K/Akt/mTOR and AMPK pathways act in contrasting fashion in metabolism and immunity. Several factors like IL-2 and CD28 signaling and growth factors activate the PI3K/Akt/mTOR pathway, resulting in survival, and proliferation of different cells. Naïve T cells use lipid β-oxidation to supply their low demands, but after activation, these cells generate a large amount of energy using glycolysis, through changing their metabolic machinery.

Levels of several metabolites [amino acids (83, 84) and glucose], growth factors, energy level (cytosolic AMP:ATP ratio), stress, and immunological signals [CD28, IL-2 (82)] regulate mTOR function (Figure 3) (83). At the same time, mTOR controls expression of several nutrition transporters (80). Different cytokines also regulate mTOR activity; IL-7 activates mTOR and inhibits autophagy, IL-4 promotes proliferation through mTOR activation and decrease apoptosis, IL-12 and IFN-γ also promote continuous mTOR activity (82).

From the immunological point of view, two signals are needed for successful activation of Th cells. The first signal is TCR recognition of antigens and the second is additional signals produced by co-stimulator molecules. If the first signal is not accompanied by the second signal, Th cells will not be able to react (anergy). Anergic T cells are metabolically oxidative. They use oxidative phosphorylation to supply their energy demands (84) and it seems that inhibition of glycolysis is sufficient for induction of anergy. For example, 2-DG which blocks glycolysis, inhibits Th17 differentiation even under Th17-polarizing conditions (13, 84). Interestingly, mTOR inhibition using Rapamycin promotes the induction of anergy in Th cells, even in the presence of second signal (82, 85). This phenomenon is explained by the fact that mTOR is downstream of the second signaling pathway, hence its inhibition attenuates the up-stream signal.

All reports suggest that mTOR is a mediator in T cells, between immunologic signals and metabolic demands. Further studies show that mTORC1 promotes Th1 and Th17 differentiation and mTORC2 induces Th2 differentiation. Suppression of both complexes results in T_reg_ induction. The effect of Rapamycin as an antibiotic and immunosuppressive medicine, which blocks mTOR activation, sheds light on an incredible connection between metabolism and immune function. Earlier studies showed that using Rapamycin and knocking out mTORC1 have similar effects (85). However, the exact mechanism of mTOR in determining Th cell fate is not well understood. It seems that mTOR plays an important role in metabolic reprograming of activated Th cells (21).

In addition to mTOR, the protein kinase AMPK also plays a critical role in metabolism and differentiation of Th cells. Similar to mTOR, AMPK is highly conserved in eukaryotic cells as well (85). AMPK and mTOR play important roles in metabolism and immunity. Their function in immunity is also against together (15, 86). By mTOR activation, glucose metabolism is promoted, especially glycolysis, and mTOR suppression by Rapamycin results in the suppression of glycolysis and corresponding increase in fatty acid oxidation (15, 36, 85). Previous studies have shown that induction of AMPK activation has similar results to the suppression of mTOR (15, 36, 85), by activation of AMPK consequently fatty acid oxidation promotes and mTOR function is suppressed. Induction of fatty acid oxidation through mTOR suppression and/or AMPK activation in activated Th cells, as mentioned above, results in T_reg_ differentiation (36, 85).

Rapamycin inhibits mTORC1 and mTORC2 function and induces T_reg_ as well. Although the inhibitory effects of Rapamycin on mTORC2 was unclear for several years, Powell, and Delgoffe in their investigation in 2010 indicated that specific doses of Rapamycin might inhibit mTORC1 and mTORC2 in T-cells (82). They claimed that Rapamycin might also promote induction of Treg cells (87).

One of the questions that arises is the possibility of induction of Th subsets *in vitro* and *in vivo* through specific metabolites in precise doses by activation and suppression of mTOR. Some previous investigations have shown this effect (88, 89). These studies help us to understand how our diet influences immune system function. Furthermore, they provide explanations for autoimmune diseases and possible key points to treat them. For example, short chain fatty acids are able to induce intestinal T_reg_ in mice. The imbalance of Th17/T_reg_ is responsible for several diseases, like inflammatory bowel diseases (IBD) (16).

Is it possible that accumulation of metabolites in the cells and excessive dietary uptake also modulate mTOR activity? Does this modulation of mTOR activity result in changes in the fate of immune responses? Both answers are yes. Not only specific metabolites like glucose (36, 90), NaCl (3), fatty acids (1, 16, 17, 91), amino acids (21), all-trans retinoic acid (18, 92), cholesterol (93, 94) and vitamins (19) affect mTOR function, but also mTOR regulates expression of metabolite transporters (84). For example, after Th cell activation, since glucose is the main source of energy for these cells, mTOR activation results in up-regulation of glucose transporters. Additionally, the presence of cytosolic leucine and glutamine is essential for mTOR function (21).

**Figure 4:**
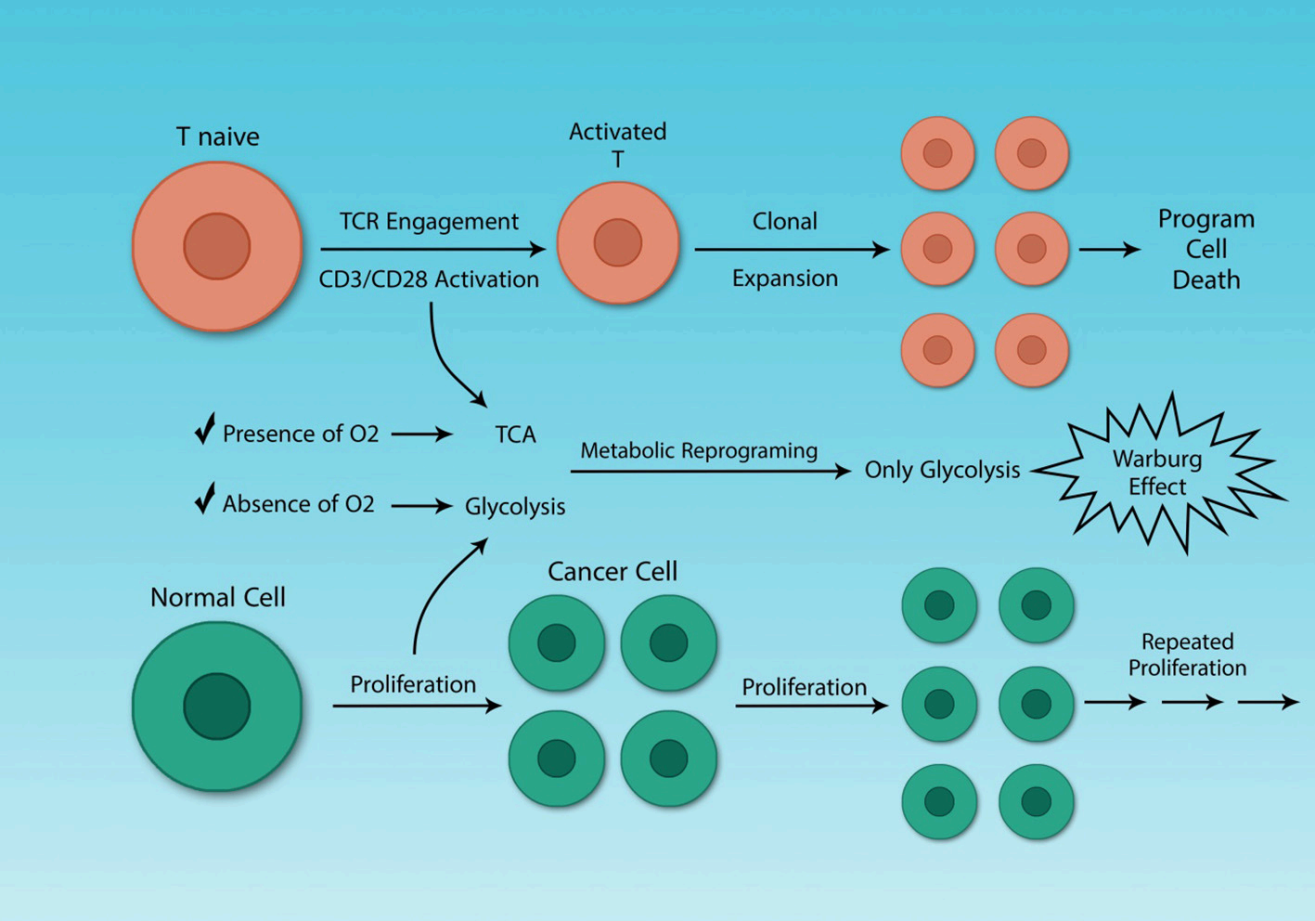
Cancer cells and activated Th cells use similar metabolic programs. Normal and cancer cells use distinct metabolic pathways, because of their different energy demands. Resting and activated Th cells also have different energy demands, because activated Th cells need to growth rapidly. Although cancer and activated Th cells are different, in general, they share a similar feature, their metabolism. The Warburg effect characterizes both cell types.

The metabolic reprogramming in activated Th cells is the same as that of cancer cells (36, 82). This similarity may seem logical, as both cells must undergo rapid proliferation and this process is highly demanding for both energy and metabolic substrates. The difference between Th cells clonal expansion and cancer cells growth is that cancer cells proliferate uncontrollably. Some molecules like mTOR play a crucial role both in the metabolism of cancer cells and activated Th cells, which has been investigated earlier (Figure 4) (82).

## Effects of Flavonoids on PI3K/Akt/mTOR Axis Based on Studies in Cancer Cells

As mentioned, cancer cells and activated Th cells have similar metabolisms. Both use glycolysis to supply their demands. Although previous studies about the effects of flavonoids on the immune system might provide some new information for nutritionists, they are incompletely understood from molecular immunology point of view. In addition, findings from these studies are heterogeneous, for instance regarding the effect of flavonoids on some inflammatory cytokines like TNF-α (23, 95–97). Detailed data are available about the impacts of different flavonoids on cancer cell proliferation. Because of metabolic similarity between cancer cells and activated Th cells and lack of sufficient data about flavonoids effects on Th cells or the PI3K/Akt/mTOR axis, here we will focus on results from the cancer field.

Flavonoids also have specific effects on this axis in cancer cells (Table 1). Curcumin, a yellow-pigment substance and component of turmeric, significantly increases mTOR suppression and induces apoptosis in renal cancer cells (105). Curcumin is also able to arrest melanoma cells in G2/M and induce autophagy in these cells. *In vitro* investigations showed that Curcumin inhibits Akt, mTOR, and P70S6K activity. Moreover, Curcumin was shown to suppress tumors in BALB/c mice, though this effect was not significant (106). In breast and prostate cancer cell lines, Curcumin inhibits Akt and mTOR function even in the presence of EGF, a ligand of the EGF receptor. The Akt/PI3K/mTOR axis is one of the most important down-stream signaling pathways after EGFR activation (107). The suppression of the Akt/PI3K/mTOR axis, even in the presence of EGF, could be a promising finding in the field of cancer therapy research. Both ligand-dependent and independent activation of EGFR can cause resistance to current therapies, a major problem in cancer treatment (108). All results confirm that Curcumin induces apoptosis and inhibits tumor cell growth and it is also able to block metastasis (101).

**Table 1:** Examples of different flavonoids targeting PI3k/Akt/mTOR pathway in different cell lines.

**Flavonoid**	**Duration**	**Cell line**	**Effect**	**References**
Fisetin	24/48 h	Lung carcinoma cell line	Inhibition of tumor cell growth Increased activation of PTEN, AMPK, and TSC2 Decreased activity of PI3K, Akt, and mTOR	(25)
Fisetin	24/48 h	Prostate cell line	Induction of apoptosis Induction of caspase 3, 8, and 9 activity Decreased activity of Cyclins, CDKs, PI3K, Akt	(98)
Gelam honey and ginger	24/48 h	Colon cell line	Inhibition of cell viability	(99)
Pomegranate	24 h	Colon cell line	mTOR, Akt and PI3k activity suppression and decreased expression	(100)
Curcumin	2 h	Colorectal cell line	Decreased mTOR and Akt expression	(101)
Quercetin	24/48 h	Prostate cell line	Suppression of mTOR and Akt activity	(102)
Baicalein	72 h	Prostate cell line	Induction of apoptosis in cancer cells mTOR and Akt activation decreased	(103)
Butein	24 h	Cervical cell line	Induction of G2/M arrest Induction of caspase 3, 8, and 9 activity Decreased activity of PI3K, Akt, and mTOR	(104)

Another attractive polyphenol, fisetin inhibits mTOR complexes, PI3K (25, 98), and Akt activity in prostate cancer cell lines (25, 98, 109). It also activates AMPK and PTEN in non-small lung cancer cells (25, 98). As mentioned before, AMPK and mTOR play contrary roles in metabolism. In cancer cells, AMPK activation and mTOR suppression result in both survival and proliferation failure (110).

Quercetin inhibits the Akt/PI3K/mTOR and Wnt/catenin pathways in lymphoma cells (111). mTOR inhibition and induction in apoptosis after Quercetin treatment in Burkitt's lymphoma cells has been observed (112). In a cervix cancer cell line, G2/M arrest was observed in the cell cycle. It also triggers release of cytochrome-C which is an indicator of apoptosis (113).

Other studies have shown that pomegranate polyphenols not only suppress Akt and mTOR expression and function, but also reduce IGF expression in colon cancer cells (100). In 2015 Zhang et al. showed that Aqueous Allspice Extract (AAE), which contains many different flavonoids, was able to activate autophagy signaling in breast cancer cells and induce cell death. AAE acts synergistically with Rapamycin and enhances autophagy and cell death. Akt and mTOR signaling are suppressed by AAE (114).

Another similar pathway that is active in tumor cells (102) as well as in Th cells (115) is aryl hydrocarbon receptor (AhR). It plays a central role in the differentiation of Th cells. If AhR is activated by dioxin or kynurenine, T_reg_ cells differentiate *in vivo* and *in vitro*, whilst its other ligand, FICZ, induces the Th17 subset (115). In prostate tumor cells, AhR shows aberrant expression and its deletion or inhibition results in the inhibition of tumorgenesis and tumor growth. By suppression of AhR, G0/G1 cell cycle arrest occurs in prostate cancer cells. It can be concluded that AhR is necessary for induction of cell cycle arrest and apoptosis by Quercetin in prostate cell line (102). However, the exact mechanism of involvement of AhR in cancer cell apoptosis mediated by Quercetin is not well understood yet. Previous studies show that AhR can activate the Akt/PI3K/mTOR pathway, and AhR inhibition results in low PI3K activity and also restores sensitivity to apoptosis in the mouse hepatoma cell line (116).

Because of similar metabolisms in active Th cells and cancer cells, described in detail above, it is expected that the polyphenols can suppress mTOR activity in Th cells. Hence, it can be concluded that polyphenols also induce T_reg_ cells and these differentiated regulatory cells suppress unwanted immune response against self-antigens.

## Conclusion

Before activation of naïve Th cells, they are catabolic. However, after activation and differentiation into effector subsets they become anabolic. If T effector cells are not able to change their metabolic status, they will be unable to respond to pathogens. The PI3k/Akt/mTOR pathway is up-regulated after Th activation, and its suppression results in anergy. By considering the important role of metabolism in the differentiation of Th cells, it seems reasonable that accumulation of specific metabolites may induce Th subsets. Indeed, flavonoids have been investigated for their effects on immune system. Flavonoids are able to modulate immune response, though the exact molecular mechanisms involved in these changes are not well understood. Flavonoids also have anti-proliferative effects on cancer cells through suppression of the PI3k/Akt/mTOR pathway in these cells. As cancer cells and activated Th cells use glycolysis and the PI3k/ Akt/mTOR pathway plays a crucial role in both cells, it can be concluded that flavonoids also suppress this pathway in Th cells. By suppression of the PI3k/AKT/mTOR pathway, T effector differentiation is reduced and T regulatory cells are induced.

## Author Contributions

AH, OS, AN, and AE contributed to design, conception and manuscript drafting. AH and OS contributed to literature search and data extraction. SS and GB contributed to manuscript drafting. All authors approved the final version of manuscript.

### Conflict of Interest Statement

The authors declare that the research was conducted in the absence of any commercial or financial relationships that could be construed as a potential conflict of interest.

## Abbreviations

AD: Atopic Dermatitis
AhR: Aryl Hydrocarbon Receptor
AMPK, AMP-activated: protein kinase
EGF: Epidermal Growth Factor
FICZ, 5, 11-Dihydroindolo 3,2: *b*carbazole-6-carboxaldehyde
IFN: Interferon
IL: Interleukin
LPS: Lipopolysaccharide
mTORC1&2: Mechanistic Target of Rapamycin Complex 1&2
PTEN: Phosphatase and Tensin homolog
T CD4^+^ or Th: T helper
T CD8^+^ or Tc: T cytotoxic
Teff: T effector cell
Treg: T regulatory cell
TCA: Tricarboxylic Acid
TNF: Tumor Necrosis Factor
2-DG: 2-deoxyglucose.
